# Mathematical energy minimization model for joining boron nitride fullerene with several BN nanostructures

**DOI:** 10.1007/s00894-021-04855-5

**Published:** 2021-08-11

**Authors:** Nawa A. Alshammari

**Affiliations:** Department of Mathematics, College of Science and Theoretical Studies, Saudi Electronic University, Riyadh, Saudi Arabia

**Keywords:** BN nanotubes, BN fullerene, BN torus, Willmore energy, Calculus of variations

## Abstract

Nanoscale materials have gained considerable interest because of their special properties and wide range of applications. Many types of boron nitride at the nanoscale have been realized, including nanotubes, nanocones, fullerenes, tori, and graphene sheets. The connection of these structures at the nanoscale leads to merged structures that have enhanced features and applications. Modeling the joining between nanostructures has been adopted by different methods. Namely, carbon nanostructures have been joined by minimizing the elastic energy in symmetric configurations. In other words, the only considerable curvature in the elastic energy is the axial curvature. Accordingly, because it has nanoscale structures similar to those in carbon, BN can also be joined and connected by using this method. On the other hand, different methods have been proposed to consider the rotational curvature because it has a similar size. Based on that argument, the Willmore energy, which depends on both curvatures, has been minimized to join carbon nanostructures. This energy is used to identify the joining region, especially for a three-dimensional structure. In this paper, we expand the use of Willmore energy to cover the joining of boron nitride nanostructures. Therefore, because catenoids are absolute minimizers of this energy, pieces of catenoids can be used to connect nanostructures. In particular, we joined boron nitride fullerene to three other BN nanostructures: nanotube, fullerene, and torus. For now, there are no experimental or simulation data for comparison with the theoretical connecting structures predicted by this study, which is some justification for the suggested simple model shown in this research. Ultimately, various nanoscale BN structures might be connected by considering the same method, which may be considered in future work.

## Introduction

Nanoscale materials have considerable interest because of their extraordinary properties and applications. In particular, carbon structures at the nanoscale such as nanotubes, nanocones, graphene sheets, tori, and fullerenes have received significant interest for application in a variety of fields, including microelectronics, sensing and actuation systems, biotechnologies, composite materials, and energy storage [12]. On the other hand, boron nitride (BN) structures at the nanoscale are distinctive because of their similarity to carbon structures at the nanoscale. Because of their remarkable properties, these particular structures have broad applications in improving nanodevices [1, 27]. The hexagonal BN structure contains boron and nitrogen atoms bound by strong covalent bonds. In addition, these nanostructures can be found in many forms, such as BN fullerene (BN fullerene), BN nanotorus (BNNTR), BN nanotube (BNNT), BN nanocone (BNNC), and BN graphene (BN graphene) forms [18, 25].

Similar to carbon nanostructures, these nanostructures have favorable thermal and mechanical properties such as high thermal conductivity, low density, high tensile strength, and membrane stiffness. Additionally, their atomic composition provides them with characteristics surpassing those of carbon nanostructures, such as stronger resistance to oxidation and chemical stability at high temperatures [12, 14, 17, 20, 23]. Furthermore, BN nanostructures have outstanding applications in energy storage, optoelectronics, electronics, biomedical medicine, and nanosemiconductor devices [13].

A BNNT can be formed as a tube structure of a hexagonal lattice involving organized boron and nitrogen atoms, and this structure was first discovered in 1994. BNNTs have an important interest among researchers because of their unique characteristics. In particular, BNNTs are electrically insulating with a band gap of 6 eV, and they are stable up to 800 °C in air. BNNTs can be considered to have outstanding thermal conductivities for high Young’s moduli above 1.3 TPa and superhydrophobicity. BNNTs are also piezoelectric and useful for spintronic devices [8].

In 1985, after the discovery of fullerene C_60_, researchers attempted to find fullerene-like compounds comprising other elements. Boron nitride fullerenes are the most attractive structures, such as B_36_ N_36_, which is quite similar to carbon fullerene C_60_. BN fullerenes have attracted much scientific research. That is, they have a higher heat resistance and a wider band gap in air than carbon structures. BN fullerenes are useful in many aspects, for example, in electronic devices, insulator lubricants, and high semiconductors. BN fullerenes that include lightweight structures are capable of storing many gas molecules per unit weight. Additionally, they are useful in gas storage, molecular sieves, or nanomembranes [16].

A boron nitride nanotorus (BNNTR) can be defined as a BN nanotube bent such that its ends are connecting together. This nanostructure has been studied in many studies based on its structure and unique properties. Different applications have been found in the literature, such as in ultrafast optical filters and nanoantennas sensitive to high-frequency electromagnetic signals [18, 30].

Connecting or joining nanostructures to other nanostructures has advantages because it leads to new nanostructures with enhanced physiochemical and electrochemical performance, affording them a broad range of uses in various fields [10, 19, 28, 32]. New joined nanoscale structures might develop the physiochemical and electrochemical performance of joined nanoscale structures, such as in nanosensors and nanooscillators. In particular, the newly joined nanoscale structures are helpful for energy storage, for designing probes for scanning tunneling microscopy, and as carriers for drug delivery [27]. Therefore, many methods have been used to join and connect nanomaterials. For example, elastic energy is employed to join different carbon nanostructures, as these nanoscale structures are supposed to deform with perfect elasticity. In particular, by minimizing the squared curvature, the Euler-Lagrange equation is obtained, which is applied to define the joint region between carbon nanomaterials. As a result of this method, many carbon nanostructures have been connected, such as carbon nanotubes and graphene, nanotubes and fullerenes, nanotubes and nanocones, fullerenes and graphenes as two fullerenes, two nanocones, nanocones and fullerenes, nanocones and fullerenes, and two parallel sheets of graphene, as detailed in [9, 11, 24] and [2]. Furthermore, another method was utilized to connect carbon nanostructures, that is, the Willmore energy [29]. This method supports the argument that considers the rotational curvature because it has a similar size. As a natural generalization of elastic energy, the Willmore energy, which involves axial curvature and rotational curvature, is used to determine the surface connection of two carbon nanoscale structures. As a result of this energy, carbon nanostructures have been joined and connected, for example, carbon nanotubes to fullerenes and two fullerenes to each other, as detailed in [29]. Furthermore, another study used this energy to join a carbon nanotorus and a nanotube [30]. In addition, similar techniques have been utilized and investigated by other researchers, for example, in [22] and [21].

On the other hand, because of their similarity to carbon in nanoscale structures, BN in nanoscale structures has been joined and connected using elastic energy. In detail, other studies have used elastic energy to connect and join BN nanostructures: BN nanocones to nanotubes and BN graphene to other BN nanostructures; and nanotubes to BN nanocones, as shown in [3] and [4], respectively. In this paper, the Willmore energy method is utilized to join and connect BN nanostructures.

The Willmore energy can be written as:

$$ \begin{array}{@{}rcl@{}} W & = {\int}_{M} H^{2} d\mu, \end{array} $$

where *H* denotes the mean curvature (which involves the sum of the rotational curvature and the axial curvature) of surface *M* and *dμ* is the area element [31]. The Willmore energy has been demonstrated to have possible applications in many different aspects of molecular, biological, and nanotechnology science. On the other hand, catenoids, as absolute minimizers of the Willmore energy, have numerous uses in nanomagnetism [5–7, 15, 26]. This paper expands the method of using catenoids to establish the conformation of the connection of BN nanostructures, namely, a BN nanotube to a BN fullerene and two BN fullerenes to each other.

In this research, we recognize that the existence of this predicted structure has not been achieved experimentally. The purpose of this research is to accommodate the major features that encapsulate the dominant physical effects so that we might imagine a real physical system in terms of departures from an ideal model. In particular, this research modeled only the mathematics of the energy inherent in the curved surface, which can be thought of as a result of chemical bonding. This model does not take into account chemical issues, such as the position of nanostructure atoms and bonds. However, the curved profiles of the graphene fold obtained by minimizing the elastic energy using calculus of variations are in excellent agreement with the experimental results [9].

In the following section, the essential equations to model the joining region between BN nanostructures are determined. In “Results”, the results are provided in three subsections, where the connection of a BN fullerene to a BN nanotube is given in “Joining BN nanotubes and fullerenes”, between two fullerenes is specified in “Joining two BN fullerenes”, and between a fullerene and torus is provided in “Joining a BN fullerene and BN nanotorus”. Finally, “Conclusions” provides the conclusion of this paper.

## Model

**Fig. 1:**
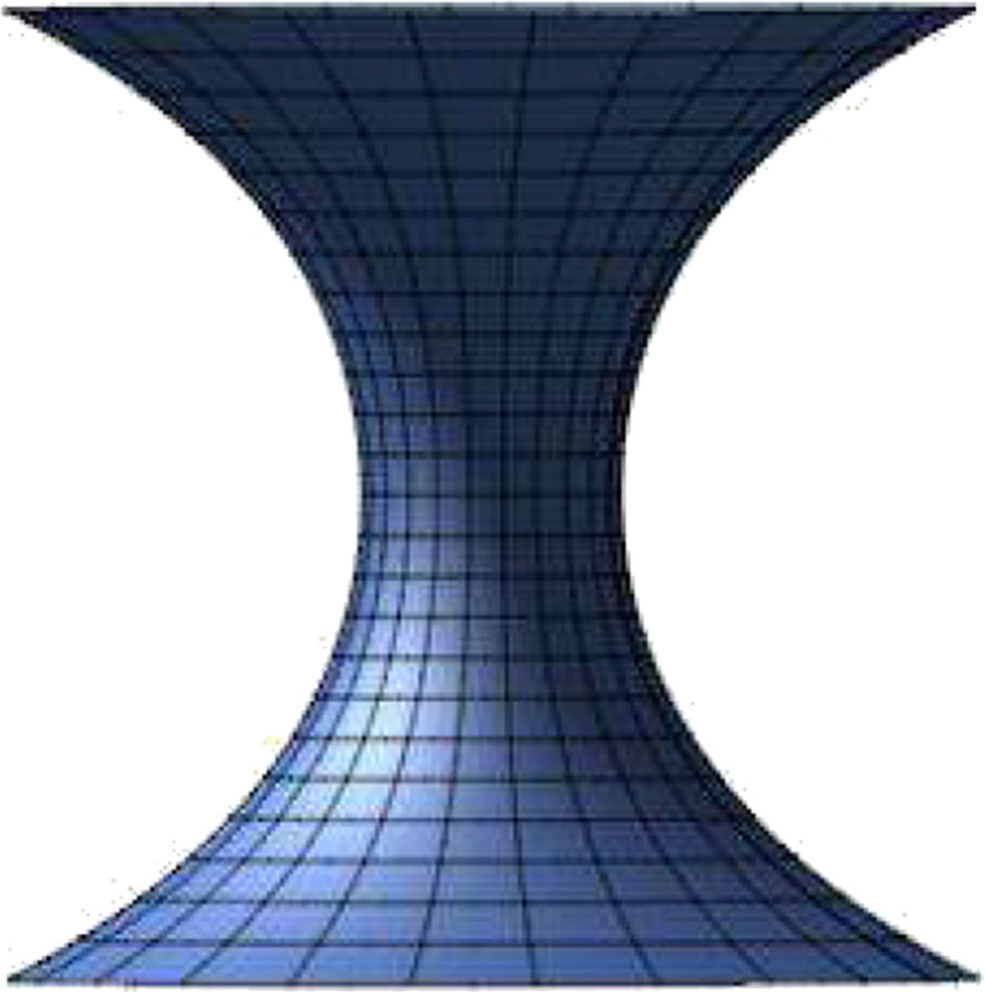
Catenoid surface

We may write the mean curvature in terms of the axial and rotational curvatures *κ*_*a*_ and *κ*_*r*_, respectively, which is called the Willmore energy function, as follows:

$$ \begin{array}{@{}rcl@{}} &J[y]=\int (\kappa_{a} + \kappa_{r})^{2} d\mu + \lambda \int d\mu, \end{array} $$

where *dμ* is an area element, *λ* is a Lagrange multiplier corresponding to an area constraint, and *H* denotes the sum of both curvatures, which is the mean curvature. Assume that the joining will have a catenoid surface $S=\{ (x,y,z): x = r {\cos \limits } \theta , y = r {\sin \limits } \theta , z = f(r)\}$, as shown in Fig. 1. The mean curvature can be written in terms of both curvatures as

$$ \begin{array}{@{}rcl@{}} &H=\kappa_{a}+\kappa_{r}=-\frac{f^{\prime\prime}(r)}{(1+f^{\prime2}(r))^{\frac{3}{2}}}-\frac{f^{\prime}(r)}{r\sqrt{f^{\prime2}(r)+1}}, \end{array} $$

if *H* = 0, this gives an absolute minimizer of the Willmore energy, the general solution might be expressed as

$$ \begin{array}{@{}rcl@{}} & f(r)= \pm \frac{\ln \left( a r +\sqrt{a^{2} r^{2} -1} \right)}{a}+ b, \end{array} $$

where *a* and *b* are arbitrary constants. In addition, this solution can be written as:

$$ \begin{array}{@{}rcl@{}} & f(r)= \pm \frac{\cosh^{-1} (a r)}{a} + b. \end{array} $$

This demonstrates the joining surface, as a catenoid, between two nanostructures. For more details of derivation, we refer the reader to [29].

In this paper, rotationally symmetric boron nitride nanostructures are joined and connected by using a part of the catenoid. In particular, we employ the boundary conditions that we have when matching the gradient at the connection points with the coordinates to find the arbitrary constants. In the following section, a catenoid is used to construct new BN nanostructures resulting from the connection between two different BN nanostructures: first, a fullerene with a nanotube; second, two fullerenes; and finally, a fullerene with a torus.

## Results

### Joining BN nanotubes and fullerenes

**Fig. 2:**
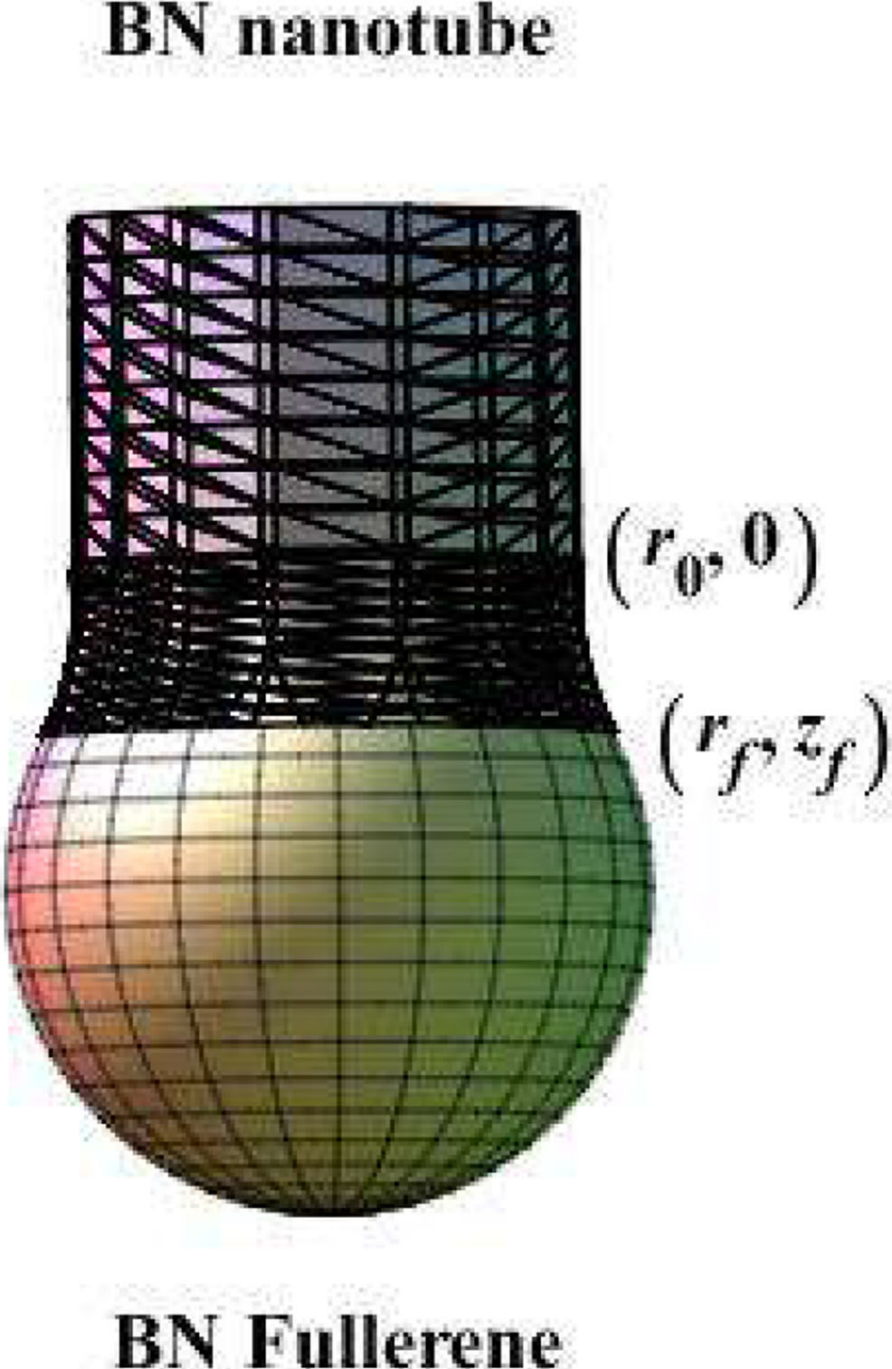
A part of the catenoid joins between the BN nanotube and BN fullerene

Figure 2 presents a three-dimensional schematic of the joined structure, while a part of the catenoid is used to join a boron nitride nanotube and a fullerene. Here, the curve of the catenoid can be written as

1 $$ \begin{array}{@{}rcl@{}} z & = -\frac{\cosh^{-1} (a r)}{a} + b, \end{array} $$

with constants *a* and *b*. Assume that the radius of the BN nanotube is the specified value *r* = *r*_*t*_. Furthermore, the fullerene equation can be expressed as

2 $$ \begin{array}{@{}rcl@{}} z & = A + \sqrt{R^{2} - r^{2}}, \end{array} $$

when the radius of the fullerene is *R* and *A* denotes the constant specifying the fullerene on the negative *z* −axis, which can be specified by the position of the connection to the catenoid. Moreover, assume that the nanotube connects the catenoid at (*r*, *z*) = (*r*_*t*_, 0) and that the other side of the catenoid connects the fullerene at (*r*, *z*) = (*r*_*f*_, *z*_*f*_). Additionally, *a*, *b*, *A*, and *r*_*f*_ are constants to be determined, where *z*_*f*_ might be known as long as we know *r*_*f*_. At the connection points and from the boundary condition, the coordinates should be consistent with the gradient. Therefore, on (*r*_*t*_,0), using Eq. 1, we obtain

$$ \begin{array}{@{}rcl@{}} b & = \frac{\cosh^{-1} (a r_{t})}{a}, \end{array} $$

and from Eq. 1, we have

$$ \begin{array}{@{}rcl@{}} \frac{dz}{dr} & = -\frac{1}{\sqrt{a^{2} r^{2} -1}}. \end{array} $$

The gradient at (*r*_*t*_, 0) gives

$$ \begin{array}{@{}rcl@{}} -\frac{1}{\sqrt{a^{2} {r_{t}^{2}} -1}} = & \infty, \end{array} $$

that is,

3 $$ \begin{array}{@{}rcl@{}} a = & \frac{1}{r_{t}}, \end{array} $$

and *b* = 0.

Additionally, on (*r*_*f*_, *z*_*f*_),

$$ \begin{array}{@{}rcl@{}} -\frac{\cosh^{-1} (a r_{f})}{a} & = A + \sqrt{R^{2} -{r_{f}^{2}}}, \end{array} $$

which gives

$$ \begin{array}{@{}rcl@{}} A & = - r_{t} \cosh^{-1} (r_{f}/r_{t}) - \sqrt{R^{2} - {r_{f}^{2}}}, \end{array} $$

matching the gradient results

$$ \begin{array}{@{}rcl@{}} -\frac{1}{\sqrt{a^{2} {r_{f}^{2}} -1}} = & \frac{r_{f}}{\sqrt{R^{2} - {r_{f}^{2}}}}, \end{array} $$

and from Eq. 3, we obtain

4 $$ \begin{array}{@{}rcl@{}} r_{f} & =\sqrt{R r_{t}}, \end{array} $$

and

5 $$ \begin{array}{@{}rcl@{}} A & = -r_{t} \cosh^{-1} \sqrt{\frac{R}{r_{t}}} - \sqrt{R(R - r_{t})}. \end{array} $$

**Fig. 3:**
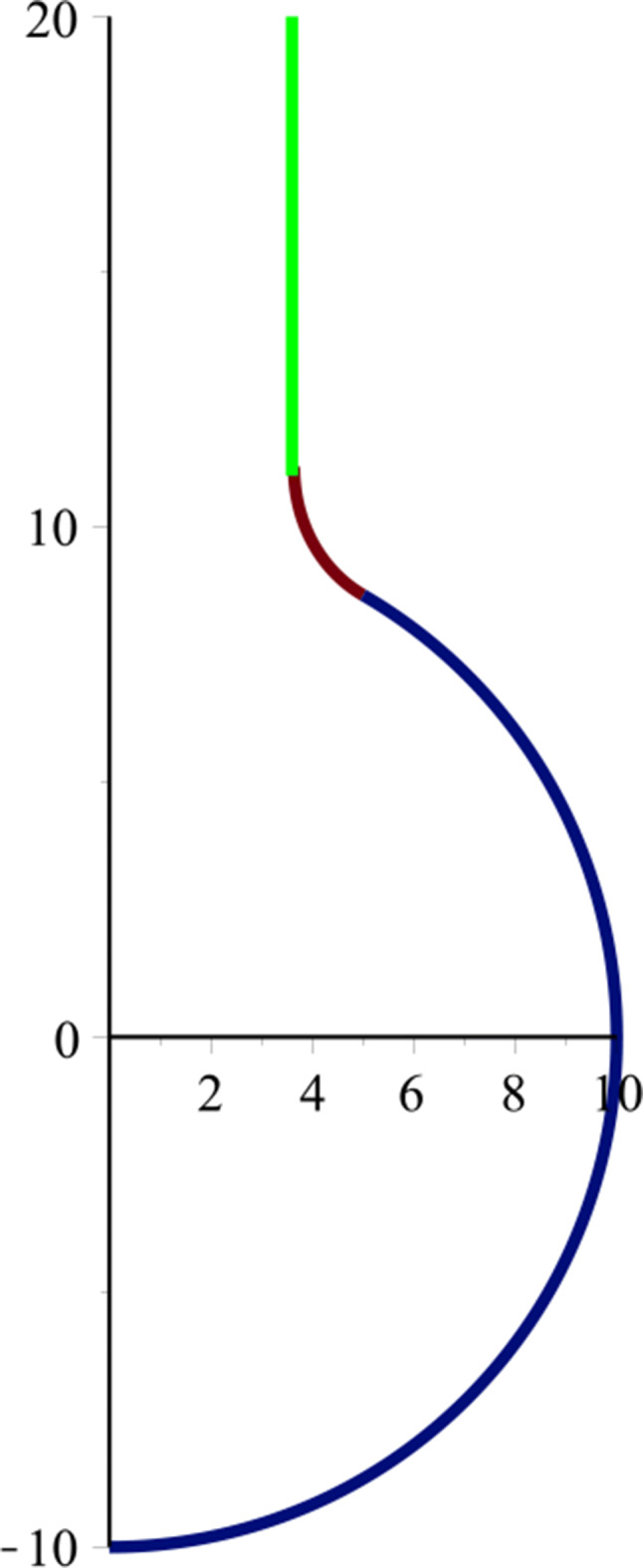
Connection profile between a BN nanotube and fullerene based on the Willmore energy

Substituting Eqs. 3, 4, and 5 to Eqs. 1 and 2, with particular values for *R* and *r*_*t*_, we obtain the connection shape between the BN nanotube and fullerene by using the catenoid curve, as shown in Fig. 3

### Joining two BN fullerenes

**Fig. 4:**
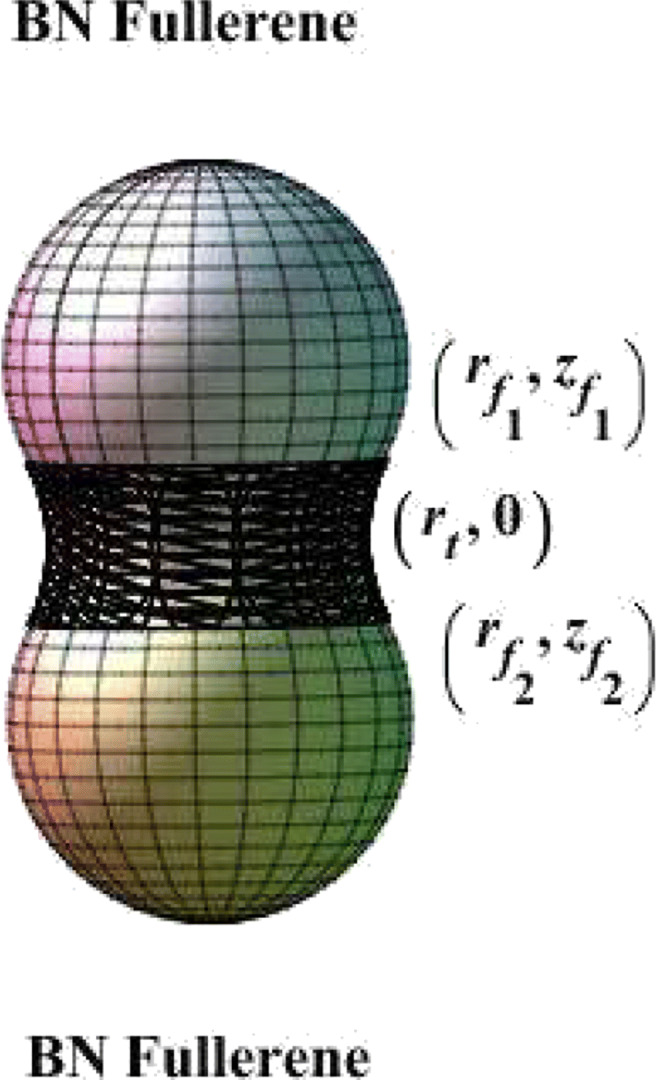
A part of the catenoid joins between two BN fullerenes

Here, the case of joining two fullerenes is considered (see Fig. 4). If we suppose the catenoid curve to be

6 $$ \begin{array}{@{}rcl@{}} z = \pm \frac{\cosh^{-1} (a r)}{a} + b, \end{array} $$

with constants *a* and *b*, then the positive sign represents the upper part of the catenoid, and the negative sign represents the lower part of the catenoid. As at the point (*r*_*t*_,0) the gradient is $\infty $, we obtain $\frac {1}{\sqrt {a^{2} {r_{t}^{2}} -1}}= \infty ,$ that is,

7 $$ \begin{array}{@{}rcl@{}} a=\frac{1}{r_{t}}, \quad b=0. \end{array} $$

Now, we assume that the upper fullerene equation is:

8 $$ \begin{array}{@{}rcl@{}} z = A_{1} - \sqrt{{R_{1}^{2}} - r^{2}}, \end{array} $$

while the radius of the upper fullerene is denoted by *R*_1_, and *A*_1_ is the constant that defines the center of the fullerene on the positive *z* −axis. Additionally, the lower fullerene can be written as

9 $$ \begin{array}{@{}rcl@{}} z = A_{2} + \sqrt{{R_{2}^{2}} - r^{2}}, \end{array} $$

where *R*_2_ denotes the radius of the lower fullerene, and *A*_2_ denotes the constant that defined the center of the fullerene with the negative *z* −axis. Afterwards, at $(r_{f_{1}}, z_{f_{1}})$ and from Eqs. 6 and 8, we obtain

10 $$ \begin{array}{@{}rcl@{}} A_{1} = r_{t} \cosh^{-1} (r_{f_{1}}/r_{t}) + \sqrt{{R_{1}^{2}} - r_{f_{1}}^{2}}. \end{array} $$

Furthermore, at point $(r_{f_{2}},z_{f_{2}})$, while the lower fullerene is connected to the lower catenoid, we have

11 $$ \begin{array}{@{}rcl@{}} A_{2} = - r_{t} \cosh^{-1} (r_{f_{2}}/r_{t}) - \sqrt{{R_{2}^{2}} - r_{f_{2}}^{2}}. \end{array} $$

Next, after matching the gradients at points $(r_{f_{1}},z_{f_{1}})$ and $(r_{f_{2}},z_{f_{2}}),$ we have

$$ \begin{array}{@{}rcl@{}} r_{f_{1}} & =& \sqrt{R_{1} r_{t}}, \\ r_{f_{2}} & =& \sqrt{R_{2} r_{t}}. \end{array} $$

As a result, we find *A*_1_ and *A*_2_ to be

12 $$ \begin{array}{@{}rcl@{}} A_{1} = r_{t} \cosh^{-1} \left( \sqrt{\frac{R_{1}}{r_{t}}}\right) + \sqrt{R_{1} (R_{1} - r_{t}) }, \end{array} $$

13 $$ \begin{array}{@{}rcl@{}} A_{2} = - r_{t} \cosh^{-1} \left( \sqrt{\frac{R_{2}}{r_{t}}}\right) + \sqrt{R_{2} (R_{2} - r_{t}) } . \end{array} $$

**Fig. 5:**
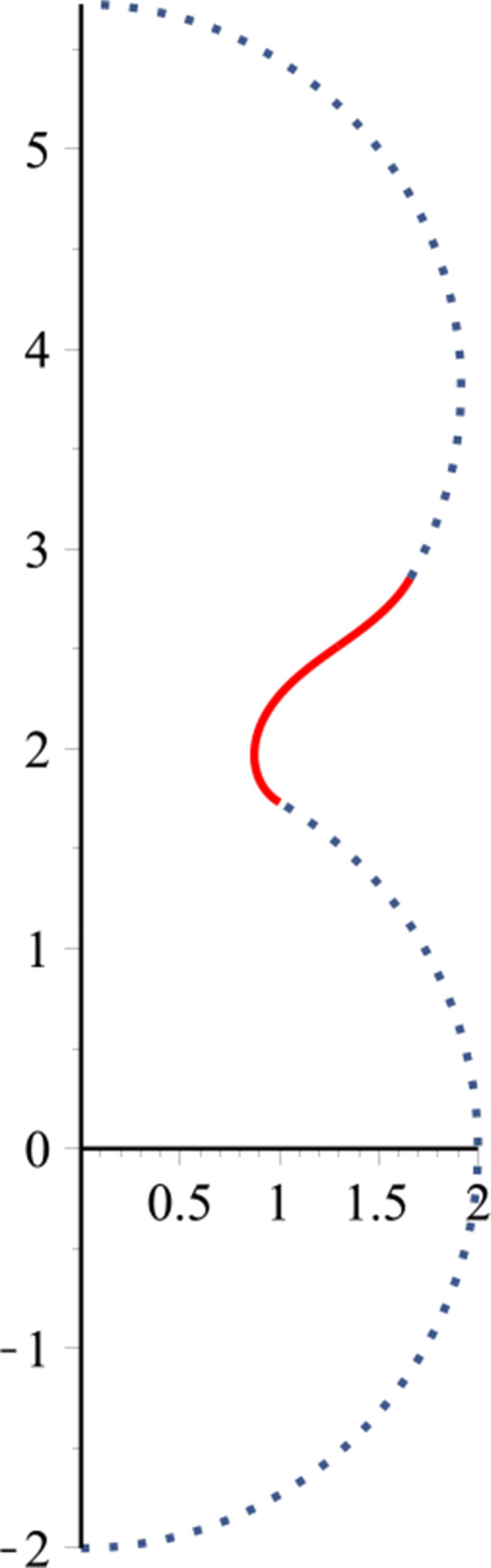
Connection profile of two BN fullerenes using the Willmore energy

By substituting Eqs. 7, 12, and 13 into Eqs. 6, 8, and 9 with specified values of *r*_*t*_ and *R*_1_ = *R*_2_, we obtain the connection shape between two fullerenes by using the catenoid curve, as shown in Fig. 5.

### Joining a BN fullerene and BN nanotorus

**Fig. 6:**
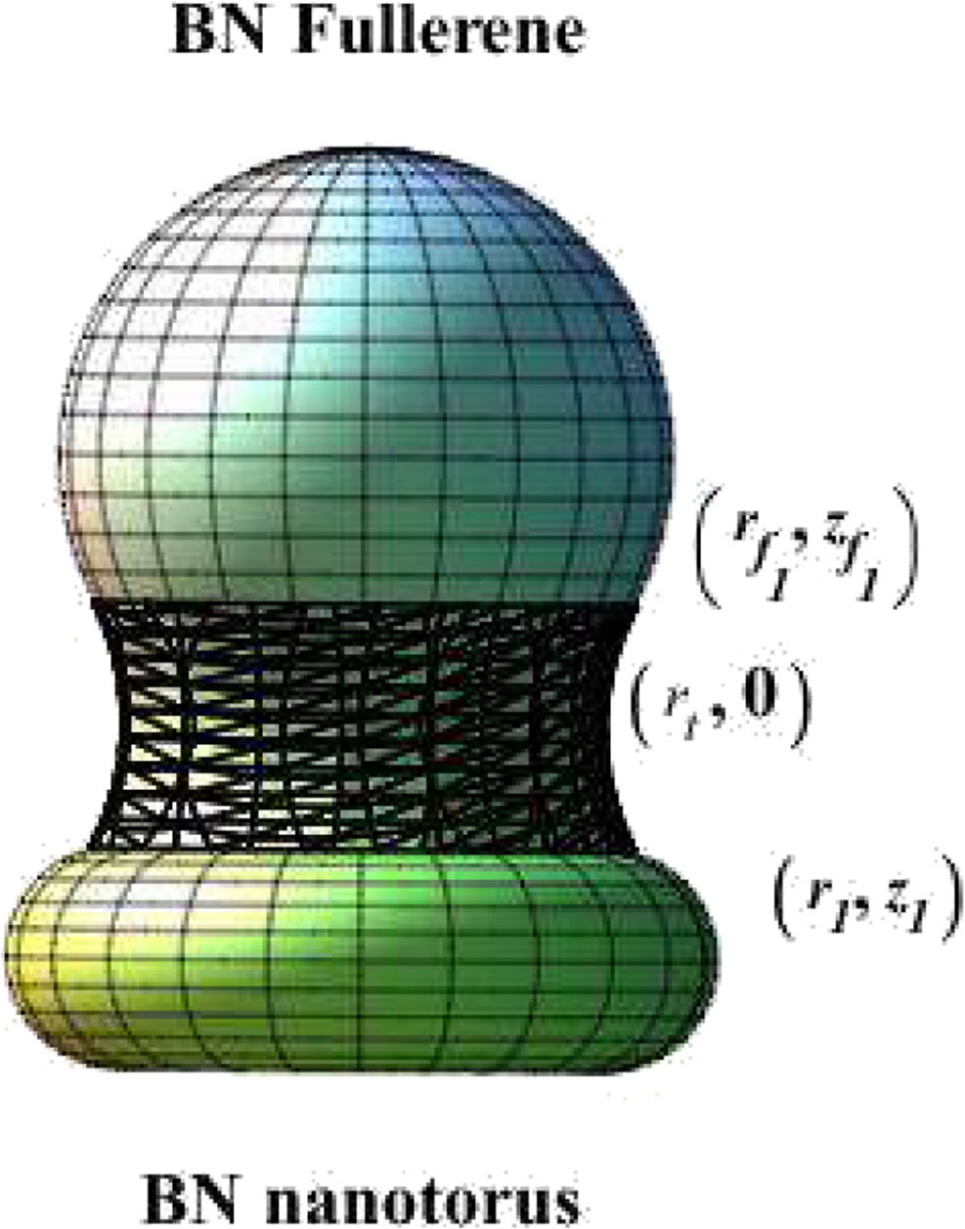
A part of the catenoid joins between a BN fullerene and nanotorus

In this case, we seek to join two different BN nanostructures, a fullerene and a torus, by using the Willmore energy, as shown in Fig. 6. We can divide this case into two parts: upper and lower parts. First, the upper part of the catenoid will join a fullerene. This case is similar to the upper part of the previous “Joining two BN fullerenes.” Therefore, we have the same calculation as this integration, and we consider Eq. 12 on this side. Second, the lower part of the catenoid will join a torus. The equation of a torus in Cartesian coordinates is

$$ \begin{array}{@{}rcl@{}} (\sqrt{x^{2} + y^{2}}-R)^{2} + z^{2}=a^{2}, \end{array} $$

and after a transformation, we obtain

$$ \begin{array}{@{}rcl@{}} x(\phi, \theta) & =& (R + a \cos \theta), \cos \theta \\ y(\phi, \theta) & =& (R + a \cos \theta), \sin \theta \\ z(\phi, \theta) & =& a \sin \theta, \end{array} $$

where *θ* and *ϕ* are polar and azimuthal angles in the *x*-axis and *xy*-plane, respectively. Additionally, *R* denotes the major radius of the torus, and *a* denotes the minor radius of the torus. Thus, the equation of the torus in cylindrical coordinates is (*r* − *R*)^2^ + *z*^2^ = *a*^2^, which can be written as

14 $$ \begin{array}{@{}rcl@{}} z & = D + \sqrt{a^{2}-(r-R)^{2}}, \end{array} $$

where *D* is a constant that can be determined by the position of the torus along the negative *z*-axis [30]. Now, at the joining point (*r*_1_, *z*_1_), considering the negative sign of Eq. 6, which represents the lower part of the catenoid, and Eq. 7 along with Eq. 14, we obtain

$$ \begin{array}{@{}rcl@{}} -\frac{\cosh^{-1}(a r_{1})}{a} &=& D + \sqrt{{a_{1}^{2}} - (r_{1}-R)^{2}},\\ D & =&-r_{t} \cosh^{-1} \left( \frac{r_{1}}{r_{t}}\right) - \sqrt{{a_{1}^{2}} - (r_{1}-R)^{2}}. \end{array} $$

Next, if we match the gradient, we have

$$ \begin{array}{@{}rcl@{}} - \frac{1}{\sqrt{a^{2} {r_{1}^{2}} -1}} & = \frac{r_{1} - R}{\sqrt{{a_{1}^{2}} - (r_{1}-R)^{2}}}, \end{array} $$

then, by substituting Eq. 7, we obtain

$$ \begin{array}{@{}rcl@{}} r_{1} = \frac{\sqrt{4 a_{1} r_{t} + R}+R }{2}, \end{array} $$

which results in

15 $$ \begin{array}{@{}rcl@{}} D &=& -r_{t} \cosh^{-1} \frac{\sqrt{4 a_{1} r_{t}+R} + R}{2 r_{t}} \\&&- \sqrt{{a_{1}^{2}}-\left(  \frac{\sqrt{4 a_{1} r_{t} + R}-R}{2} \right)^{2}}. \end{array} $$

**Fig. 7:**
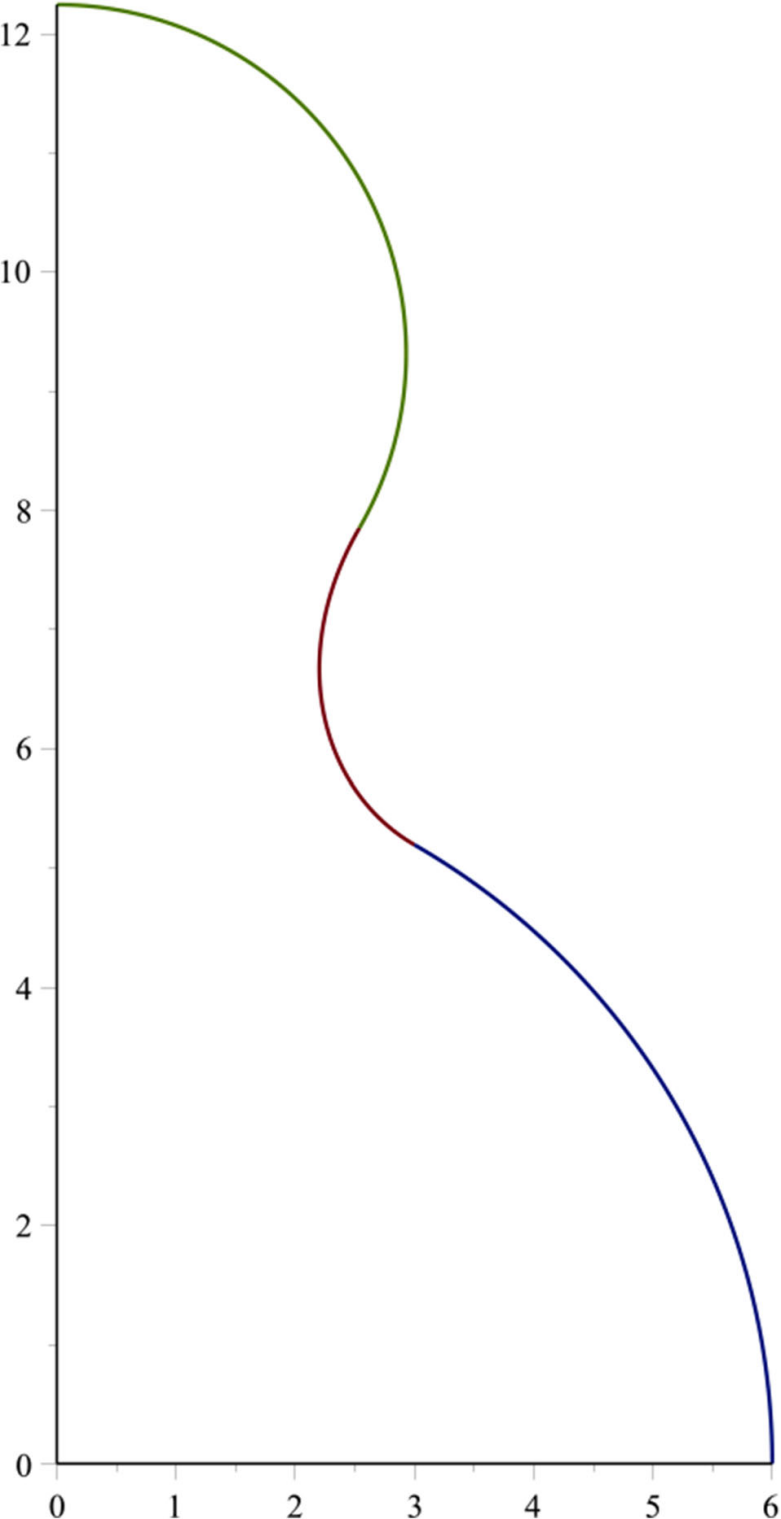
Connection profile of a BN fullerene and nanotorus by using the Willmore energy

Now, by substituting Eqs. 7, 12 and 15 into Eqs. 6, 8 and 14, respectively, with specified values of *r*_*t*_, *R*_1_, *a*_1_, and *R*, we obtain the joining shape between the fullerene and torus by using the catenoid curve, as shown in Fig. 7.

## Conclusions

In conclusion, calculus of variations is utilized to determine the connection scenarios among boron nitride nanostructures. Minimizing the elastic energy to define the connection area between carbon nanostructures has been used in many studies, while the problem is considered to be in a two-dimensional *xy* −plane. In addition, this method has been applied in the literature to join boron nitride nanostructures because of their similarity to carbon nanostructures. Furthermore, minimizing the Willmore energy, which is an extension of the elastic energy, can specify the joining curve between carbon nanostructures, while in this case, this problem is treated as a three-dimensional problem. In particular, a part of the catenoid has been used in this setting because it is a minimal energy surface. In this research, the Willmore energy minimization method is extended to cover the joining scenarios of other nanomaterials, that is, boron nitride structures at the nanoscale. The results of this method are provided for three different joining structures, namely, a BN nanotube and BN fullerene, a BN fullerene and BN fullerene, and a BN fullerene and BN nanotorus. Note that all the shapes used are assumed to be geometrically perfect structures and have not been deformed from the original shapes. The main aim in this study is to formulate the underlying axially symmetric model so that we have a reference basis for the comparison of real physical structures. Although there are no experimental or computational results for comparison, simple models appear to give rise to meaningful approximations to complex structures and therefore might be useful for future work on this problem. Ultimately, various nanoscale BN structures might be connected by considering the same method, which may be considered future work.
